# Chromosome Territories Meet a Condensin

**DOI:** 10.1371/journal.pgen.1002939

**Published:** 2012-08-30

**Authors:** Tatsuya Hirano

**Affiliations:** Chromosome Dynamics Laboratory, RIKEN Advanced Science Institute, Wako, Saitama, Japan; The University of North Carolina at Chapel Hill, United States of America

In human diploid cells, 23 pairs of chromosomes become recognizable as a discrete set of rod-shaped entities during the mitotic stage of the cell cycle. Upon exit from mitosis, they decondense and are packed together into the nucleus, in which individual chromosomes are no longer discernible. Nonetheless, evidence accumulated during past decades suggests that the decondensed chromosomes are not randomly located within the nucleus, but instead occupy distinct subnuclear domains known as chromosome territories (CTs) [1]. This raises a number of obvious questions. What protein factor(s) is(are) involved in the formation of CTs? Is there any mechanistic link between mitotic chromosome structures and interphase chromosome territories? In this issue of *PLOS Genetics*, Bauer et al. provide a remarkable answer to these questions by demonstrating that condensin II, one of the major chromosome condensation factors in mitosis [2], also makes an important contribution to the formation of chromosome territories during interphase [3].

To address the question of how interphase chromosomes might be organized, the authors focused on nurse cells in the ovary of the fruit fly *Drosophila melanogaster*, which display a highly characteristic mode of chromosome organization and dynamics. The chromosomes in these cells are amplified by repeated rounds of DNA replication without intervening mitoses (i.e., endoduplication), giving rise to polytene chromosomes in which many copies of homologous chromosomes and chromatids are paired along their entire lengths. After the fifth cycle of endoduplication, however, the pairing of homologous chromatids is disrupted, allowing each set of chromosome arms to occupy a discrete globular territory that is reminiscent of a CT observed in mammalian cells. A previous study from the same group demonstrated that condensin II function is required for the disassembly of polytene chromosomes occurring at this stage [4]. In the current study, Bauer et al. followed the fate of these chromosomes further and found that CTs are not properly formed in the condensin II mutants [3]. The authors also noticed that chromosomes in these mutants maintain their initial orientation (known as the Rabl configuration) in which the centromeric regions of all chromosomes cluster near the nuclear periphery and the telomeres cluster near the opposite pole of the nucleus (Figure 1A).

**Figure 1:**
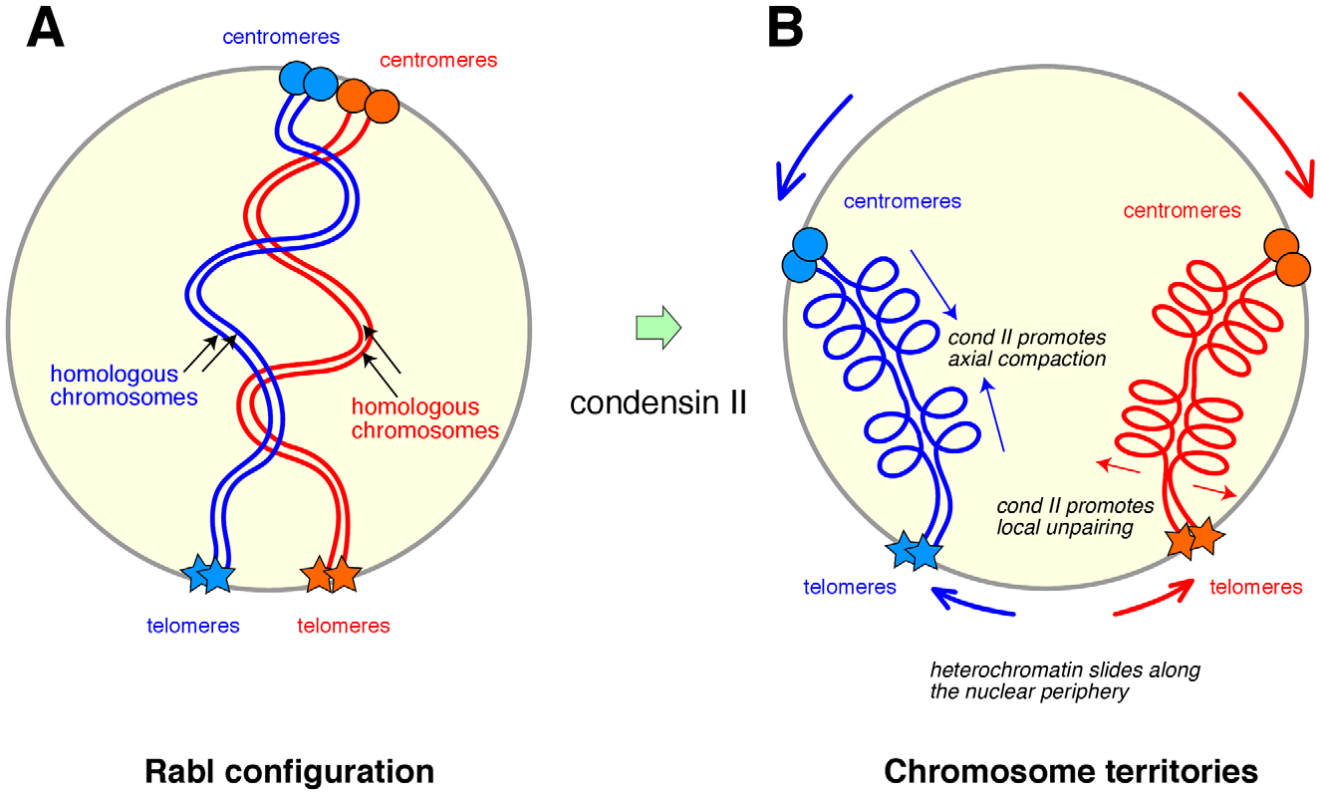
A model for how condensin II might contribute to the formation of chromosome territories in diploid cells. (A) After mitosis, decondensed chromosomes adopt the Rabl configuration, in which the centromeric regions (circles) of all chromosomes cluster near the nuclear periphery and the telomeres (stars) cluster near the opposite side of the nucleus. This configuration reflects the orientation of chromosomes in previous anaphase. In *Drosophila* diploid cells, homologous chromosomes pair along their lengths. For simplicity, only one arm of each chromosome is drawn here. (B) According to the model proposed by Bauer at al. [3], condensin II promotes axial compaction of the chromosome arms. Coincidentally, centromere clustering is dissolved, allowing heterochromatic regions (represented by the centromeres and telomeres) to slide along the nuclear periphery. As a natural consequence of these two conditions, each pair of homologous chromosomes is sequestered into a discrete chromosome territory. Intrachromosomal folding mediated by condensin II helps disrupt interchromosomal interactions, thereby promoting unpairing of homologous chromosomes and antagonizing transvection. The basic idea presented here could potentially be applied to diploid cells in other organisms where the pairing of homologous chromosomes is not necessarily observed.

Why, then, do chromosomes fail to form CTs when condensin II function is impaired? An extensive set of fluorescence in situ hybridization (FISH) data suggested that condensin II promotes not only axial compaction of chromosomes, but also dispersal of heterologous centromeres. Importantly, the dispersed pericentromeric heterochromatin nonetheless stayed near the nuclear periphery. Conversely, when one of the condensin II–specific subunits (Cap-H2) was overexpressed in salivary glands, their polytene chromosomes were disassembled, leading to axial shortening of chromosomes and CT formation. Based on these data, the authors propose a simple and appealing model in which the combination of two events, condensin II–mediated axial compaction and sustained interactions of heterochromatic regions with the nuclear periphery, drives each chromosome into a globular CT (Figure 1B). They also suggest that intrachromosomal folding promoted by condensin II disfavors interchromosomal interactions, thereby contributing to the disassembly of polytene chromosomes.

Although this is an interesting and plausible model, one could ask to what extent the insights obtained from the highly specialized polytene nuclei might be applicable to diploid nuclei. In *Drosophila*, unlike in other metazoans, homologous chromosomes are always paired in virtually all types of cells throughout development. It has long been known that the pairing of homologous chromosomes affects gene expression at a number of loci through a process known as transvection [5]. Notably, previous studies showed that condensin II antagonizes transvection in the imaginal wing disc [4], and also contributes to CT formation in diploid spermatocytes during meiosis [6]. Moreover, a more recent genome-wide screen identified condensin II subunits as factors that antagonize the pairing of homologous chromosomes in *Drosophila* tissue culture cells [7]. It will be of great importance to test whether condensin II–mediated axial shortening of chromosomes indeed underlies all these processes, although measurements of chromosome lengths are technically more challenging in diploid cells than in polyploid cells.

The outcome of these emerging studies strongly implies that chromosome–chromosome interactions in *cis* (i.e., intrachromosomal folding) and those in *trans* (interchromosomal pairing) compete with each other. Interestingly, a similar principle has already been put forward to delineate the dynamic interaction between sister chromatids within a duplicated chromosome. Upon entry into mitosis, bulk cohesin is lost from chromatid arms, and condensin II coincidentally initiates intrachromatid folding. As a consequence, sister–sister interactions are loosened, leading to the formation of metaphase chromosomes in which rod-shaped sister chromatids are juxtaposed along their lengths, a process known as sister chromatid resolution [8]. A recent study using *Xenopus laevis* egg extracts has demonstrated that this process is indeed controlled by an intricate balance between the cohesin-mediated cohesive forces and condensin II–mediated resolving forces [9]. Moreover, condensin II has been shown to contribute to axial compaction of chromosomes during mitosis in *Xenopus* egg extracts [9] and in chicken DT40 cells [10], implying that condensin II can perform essentially the same job during interphase and mitosis.

In summary, a unique and powerful experimental system, combined with a labor-intensive assay, enabled Bauer et al. to propose a model of how CTs might be formed in *Drosophila* interphase nuclei [3]. To what extent the current model might be applicable to organisms with more complex genomes remains to be determined. It is unlikely that condensin II is the sole factor contributing to CT formation. Searches for additional factors in different model systems would help advance our understanding of the organization of interphase chromosomes, and computational simulations could complement such efforts (e.g., [11]). The current work also reinforces the role of condensin II as one of the central organizers of chromosomes from interphase through mitosis. Future studies should be aimed at a mechanistic understanding of the fundamental principles of chromosome architecture and dynamics throughout the cell cycle as well as during the development of an organism.

## References

[1] Cremer T, Cremer M, Dietzel S, Müller S, Solovei I, et al (2006) Chromosome territories–a functional nuclear landscape. Curr Opin Cell Biol 18: 307–316. 16687245 10.1016/j.ceb.2006.04.007

[2] Hirano T (2012) Condensins, universal organizers of chromosomes with diverse functions. Genes Dev 26: 1659–1678. 22855829 10.1101/gad.194746.112 PMC3418584

[3] Bauer CR, Hartl TA, Bosco G (2012) Condensin II promotes the formation of chromosome territories by inducing axial compaction of polyploid interphase chromosomes. PLoS Genet 8: e1002873 doi:10.1371/journal.pgen.1002873. 22956908 10.1371/journal.pgen.1002873 PMC3431300

[4] Hartl TA, Smith HF, Bosco G (2008) Chromosome alignment and transvection are antagonized by condensin II. Science 322: 1384–1387. 19039137 10.1126/science.1164216

[5] Duncan IW (2002) Transvection effects in Drosophila. Annu Rev Genet 36: 521–556. 12429702 10.1146/annurev.genet.36.060402.100441

[6] Hartl TA, Sweeney SJ, Knepler PJ, Bosco G (2008) Condensin II resolves chromosomal associations to enable anaphase I segregation in Drosophila male meiosis. PLoS Genet 4: e1000228 doi:10.1371/journal.pgen.1000228. 18927632 10.1371/journal.pgen.1000228 PMC2562520

[7] Joyce EF, Williams BR, Xie T, Wu C-T (2012) Identification of genes that promote or antagonize somatic homolog pairing ssing a high-throughput FISH-based screen. PLoS Genet 8: e1002667 doi:10.1371/journal.pgen.1002667. 22589731 10.1371/journal.pgen.1002667 PMC3349724

[8] Shintomi K, Hirano T (2010) Sister chromatid resolution: a cohesin releasing network and beyond. Chromosoma 119: 459–467. 20352243 10.1007/s00412-010-0271-z

[9] Shintomi K, Hirano T (2011) The relative ratio of condensin I to II determines chromosome shapes. Genes Dev 25: 1464–1469. 21715560 10.1101/gad.2060311 PMC3143936

[10] Green LC, Kalitsis P, Chang TM, Cipetic M, Kim JH, et al (2012) Contrasting roles of condensin I and II in mitotic chromosome formation. J Cell Sci 125: 1591–1604. 22344259 10.1242/jcs.097790 PMC3336382

[11] Cook PR, Marenduzzo D (2009) Entropic organization of interphase chromosomes. J Cell Biol 186: 825–834. 19752020 10.1083/jcb.200903083 PMC2753166

